# Development of an EMG-Controlled Knee Exoskeleton to Assist Home Rehabilitation in a Game Context

**DOI:** 10.3389/fnbot.2019.00067

**Published:** 2019-08-27

**Authors:** Mingxing Lyu, Wei-Hai Chen, Xilun Ding, Jianhua Wang, Zhongcai Pei, Baochang Zhang

**Affiliations:** ^1^School of Automation Science and Electrical Engineering, Beihang University, Beijing, China; ^2^Department of Health Sciences and Technology, ETH Zurich, Zurich, Switzerland; ^3^College of Electrical Engineering and Automation, Shandong University of Science and Technology, Qingdao, China; ^4^School of Mechanical Engineering and Automation, Beihang University, Beijing, China

**Keywords:** electromyography (EMG), game context, home rehabilitation, human-computer interaction, Kalman filter, knee exoskeleton, stroke

## Abstract

As a leading cause of loss of functional movement, stroke often makes it difficult for patients to walk. Interventions to aid motor recovery in stroke patients should be carried out as a matter of urgency. However, muscle activity in the knee is usually too weak to generate overt movements, which poses a challenge for early post-stroke rehabilitation training. Although electromyography (EMG)-controlled exoskeletons have the potential to solve this problem, most existing robotic devices in rehabilitation centers are expensive, technologically complex, and allow only low training intensity. To address these problems, we have developed an EMG-controlled knee exoskeleton for use at home to assist stroke patients in their rehabilitation. EMG signals of the subject are acquired by an easy-to-don EMG sensor and then processed by a Kalman filter to control the exoskeleton autonomously. A newly-designed game is introduced to improve rehabilitation by encouraging patients' involvement in the training process. Six healthy subjects took part in an initial test of this new training tool. The test showed that subjects could use their EMG signals to control the exoskeleton to assist them in playing the game. Subjects found the rehabilitation process interesting, and they improved their control performance through 20-block training, with game scores increasing from 41.3 ± 15.19 to 78.5 ± 25.2. The setup process was simplified compared to traditional studies and took only 72 s according to test on one healthy subject. The time lag of EMG signal processing, which is an important aspect for real-time control, was significantly reduced to about 64 ms by employing a Kalman filter, while the delay caused by the exoskeleton was about 110 ms. This easy-to-use rehabilitation tool has a greatly simplified training process and allows patients to undergo rehabilitation in a home environment without the need for a therapist to be present. It has the potential to improve the intensity of rehabilitation and the outcomes for stroke patients in the initial phase of rehabilitation.

## 1. Introduction

Stroke is a major cause of chronic motor disability among adults worldwide (Feigin et al., 2009; Langhorne et al., 2009, 2011). Many stroke survivors suffer from hemiplegia, which makes walking difficult or even impossible.

Neurorehabilitation training has been widely used to reduce the handicap and disability caused by stroke (Langhorne et al., 2011). Recent studies have shown that a unique time-limited window of enhanced neuroplasticity for 1–3 months exists after ischemic stroke (Zeiler and Krakauer, 2013), known as the post-stroke sensitive period. Within this unique critical period, both spontaneous and intervention-mediated recovery from impairment are maximal (Murphy and Dale, 2009; Floor et al., 2013; Zeiler and Krakauer, 2013). Motor training and enriched rehabilitation during this period are especially effective in enhancing muscle activity and improving neuromuscular control. However, a crucial question remains regarding how to take best advantage of this critical time-limited window. One major problem is that patients cannot make overt movements, although they may regain some muscular control ability early after a stroke (Lyu et al., 2017). This barrier greatly limits the delivery of motivational rehabilitation training to patients.

One possible way to overcome this obstacle is the use of a “muscle–computer interface,” which tests the electromyographic (EMG) activity of the patient and provides feedback. As an easy-to-use tool, EMG signals have been successfully applied to powered exoskeletons (Tucker et al., 2015; Long et al., 2016; Lambelet et al., 2017). The critical advantage of EMG-based methods is that even though the human subject is unable to generate sufficient joint torque, their intention can still be detected from residual EMG activity and consequently the exoskeleton can be controlled (Peternel et al., 2016). It is therefore possible to train patients during the post-stroke sensitive period.

Different EMG-based exoskeletons have been developed in the past few decades. Several studies have estimated muscular torques from EMG activity using a musculoskeletal model, and this approach has been applied to the control of both upper limb (Buongiorno et al., 2016) and lower limb (Long et al., 2016; Ao et al., 2017) exoskeletons. As alternatives to a musculoskeletal model, some researchers have proposed the use of neural networks to learn the complex relation between EMG and muscular torque (Song and Tong, 2005; Chen X. et al., 2017) or of statistical learning algorithms to classify different action modes or motion patterns from measured EMG signals (Irastorza-Landa et al., 2017; Yun et al., 2017).

However, most of these approaches were designed for use in rehabilitation centers or clinics with the assistance of therapists, leading to greatly increased costs and limiting the intensity of rehabilitation treatment (Chen J. et al., 2017). There are several reasons for the limited application scenarios of these exoskeletons. First, the high cost of traditional EMG acquisition equipment and the complex electrode placement procedure required make it unsuitable for home rehabilitation (Hakonen et al., 2015). Since the signal-to-noise ratio can be improved by placing the electrodes as close to the EMG source as possible (Hakonen et al., 2015), the electrodes of standard EMG laboratory equipment are designed to be placed separately on the skin overlying specific muscles. However, the selections of which muscles to use and the positioning of the electrodes usually need to be done by the therapist. Besides, skin preparation, which is usually needed for traditional EMG electrodes, and accurate placement are time consuming as well (Cram and Rommen, 1989; Marquez et al., 2018). The cost of EMG equipment is also too high for a patient undergoing home rehabilitation. The second reason relates to the control methods. Both the musculoskeletal model and the neural network method expend most effort on increasing the accuracy of predicting muscle torque or in classification, which are important with regard to biomechanics and physiology (Lenzi et al., 2012). However, these methods depend strongly on the subject's anatomy as well as on the placement of the electrodes and usually require a precise calibration procedure, which may be unnecessary for effective exoskeleton control (Lenzi et al., 2012). User-dependent and session-dependent calibration procedures are time-consuming and cannot be done by the subject alone, which limits their use to a laboratory environment rather than a home setting. Moreover, the inconvenience of donning and removing the EMG sensor and exoskeleton, the complex setup procedure, and the boring training process also make these exoskeletons unsuitable for home-based rehabilitation. Thus, the development of an EMG-controlled exoskeleton that is simple, acceptable, and effective in improving lower limb function and is able to assist home rehabilitation for patients in the post-stroke sensitive period is an urgent task.

The challenges of home-based robotic therapy are to make the rehabilitation robot system safe and easy to use in a home setting (Sivan et al., 2014). The rehabilitation system should be acceptable to the patient and enable them to complete the training process independently, without the therapist being present for each session. The technology also needs to match general therapy principles (e.g., intensity, motivation) and provide the patient with the relevant therapy (Sivan et al., 2014). For an EMG-controlled exoskeleton, choosing the optimal way to assemble the electrodes, making it easy to don and remove the EMG sensor and exoskeleton, simplifying the calibration and setup procedures, developing appropriate methods to process the EMG signal, and maintaining motivation to participate in rehab are all of significant importance.

There have been a few studies investigating simple solutions for providing effective robotic assistance by exoskeletons (Lenzi et al., 2012; Lince et al., 2017). Lenzi et al. (2012) modeled the EMG–torque relationship by a second-order Butterworth filter and applied an assistive torque proportional to the envelope of the EMGs to an elbow exoskeleton. Their study showed that subjects can compensate for the imprecision of torque estimates and still benefit from robotic assistance. This approach has the advantage that the proportional control greatly decreases the complexity of setup, but it also suffers from the filtering method used, which is unable to maintain smoothness and responsiveness. Menegaldo (2017) found that the delay caused by the Butterworth filter can be up to 320 ms, which is too long to allow real-time control. Compared with a Butterworth filter, a Kalman filter reduced both the delay and the computing demand remarkably (Menegaldo, 2017). Some passive training devices like robots with continuous passive motion (CPM) were also performed in the home setting (Lynch et al., 2005; Hu et al., 2009; Mau-Moeller et al., 2014).

The study aims at improving the effectiveness of stroke rehabilitation in the initial phase. Our efforts focus on delivering intensive and motivational rehabilitation training to these patients. To improve training intensity, realizing home rehabilitation with exoskeleton is definitely helpful, since it makes rehabilitation easier to access. In order to motivate the patients, we first choose EMG control to involve the patient's neural system into the rehabilitation. Second, biofeedback is provided to the patient so that they can easily observe their muscle activities. Third, a game is further developed to make the training process more interesting and challenging. This study contributes to making home rehabilitation accessible for the stroke patients at the critical period, since most patients went home after 29–55 days in hospital (Jørgensen et al., 1995).

In the present study, we develop an EMG-controlled knee exoskeleton to assist home rehabilitation and investigate whether healthy subjects can use it to perform a challenging task and further improve their control strategy after practicing a visuomotor game. This user-friendly training system, which can be applied to both stroke patients and healthy subjects, provides a motivating and challenging training environment. Based on this setup, we investigate whether training with an EMG-controlled knee exoskeleton in a game context can provide significant motor learning in healthy subjects.

As compared with passive training in the home setting like a CPM device (Lynch et al., 2005; Hu et al., 2009; Mau-Moeller et al., 2014), since training of the proposed system can be performed actively, high effects should also be expected in the functional recovery. Game contexts developed in this study are also good for continuous training. Taking together, significant differences are expected in the proposed system.

This paper describes the EMG sensor and the data processing method, as well as the mechanical design and control strategy of the exoskeleton for home-based therapy. A pilot experiment on six healthy subjects establishes the feasibility of the training system. Results concerning both the evaluation of the system and the performance of the experimental subjects are presented and discussed, and indicate that this home-used rehabilitation tool shows promise for improving the outcomes for stroke patients in the initial phase of rehabilitation.

## 2. Materials and Methods

Six healthy subjects (four males and two females, mean age 24 years, range 22–26 years) who were naive to this training system were recruited to the experiment. The study was approved by the Biological and Medical Ethics Committee of the Beijing University of Aeronautics and Astronautics in accordance with the Declaration of Helsinki, and all subjects gave written informed consent before participation.

**Figure 1:**
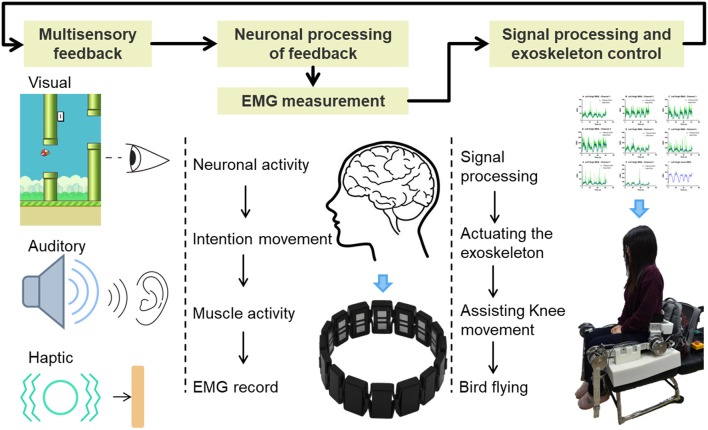
Closed-loop rehabilitation process with the proposed training system.

EMG activity of the thigh muscles was recorded by a Myo thigh-band (Figure 1) and then processed by a Kalman filter to use in controlling an exoskeleton. The lower limb exoskeleton, which has four active degrees of freedom (DOF), was seated on a chair and used to assist the subject in knee rehabilitation. A Flappy Bird game, which was implemented in Python 2.7 on a standard computer with Ubuntu 14.04.03 operating system, was used to motivate the subject to do active training, with the bird stimulated by the knee joint of the exoskeleton. During the training process, the subject was seated on the chair wearing the Myo thigh-band and the exoskeleton. In the game context, the subject needed to keep the flappy bird flying across a series of pipes and obtain as high a score as possible by trying to extend their shank against gravity to control the movement of the knee exoskeleton. Multisensory simulation was provided to the subject. This setup is intended to facilitate strengthening of anti-gravity knee extensor muscles and improving knee joint movement stability and accuracy.

### 2.1. The EMG Sensor

The Myo thigh-band (Figure 1), which was reassembled from two Myo armbands (Thalmic Labs Inc., www.myo.com), consists of 16 dry surface EMG (sEMG) sensors and two nine-axis inertial measurement units (IMUs) as well as two vibrating motors. The thigh-band electrodes form an extendable cuff that is able to adjust to the thigh in a flexible manner. A subject is able to don and remove the thigh-band without the therapist needing to be present. Vibrating motors are used to provide haptic feedback, which is applied during the game play (described in more detail below). With a sampling frequency of 200 Hz for raw sEMG data, the Myo thigh-band communicates wirelessly with the host computer via Bluetooth Low Energy (BLE).

### 2.2. Data Processing of the EMG

The “raw” EMG data from Myo thigh-band, which have been rectified and low-pass filtered, are still quite noisy and cannot be used directly. Traditional filtering methods like moving average windowing (Lee et al., 2011; Chen and Wang, 2013) and the Butterworth filter (Lenzi et al., 2012) have relatively long time lags that make them unsuitable for real-time control, especially in the challenging game context. Here a Kalman filter is used to process the acquired raw EMG data.

**Figure 2:**
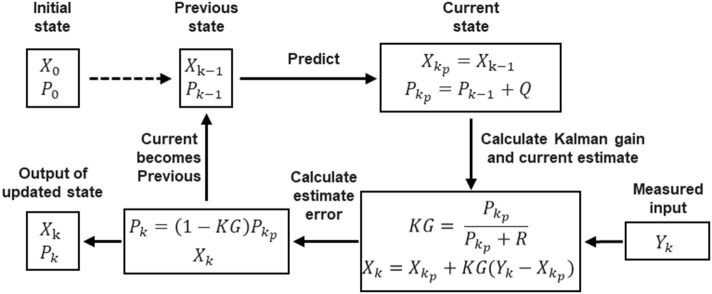
Block diagram of the Kalman filter, which involves four steps: (1) predicting current state; (2) calculating Kalman gain and producing current estimate; (3) calculating estimate error; (4) updating state.

Denoting the measured raw EMG by *Y*_*k*_ and the filtered EMG by *X*_*k*_, we initialize the previous estimate *X*_*k*−1_ as *X*_0_ and its estimated error *P*_*k*−1_ as *P*_0_. As depicted in Figure 2, the process of using the Kalman filter to estimate EMG can be divided into four steps. The first is the prediction step, in which the Kalman filter produces an estimate of the current state variable *X*_*k*_*p*__, along with its uncertainty or error in estimate *P*_*k*_*p*__ according to the previous states *X*_*k*−1_ and *P*_*k*−1_:

(1) $$X_{k_{p}}\;=\;X_{k\;-\;1}$$

(2) $$P_{k_{p}}\;=\;P_{k\;-\;1}\;+\;Q$$

Here we assume that the EMG estimate does not change for each time step, so the prediction *X*_*k*_*p*__ is the same as the previous state *X*_*k*−1_. In Equation (2), the process noise variance *Q* is added to the estimate error *P*_*k*_*p*__.

Once the next measurement *Y*_*k*_, which is corrupted with measurement noise variance *R*, has been observed, the estimate is updated using a weighted average (Kalman gain *KG*):

(3) $$KG\;=\;P_{k_{p}}/\left(P_{k_{p}}\;+\;R\right)$$

(4) $$X_{k}\;=\;X_{k_{p}}\;+\;KG(Y_{k}\;-\;X_{k_{p}})$$

The greater the certainty of the estimation, the larger is the Kalman gain. *X*_*k*_ is the output of the Kalman filter, namely, the filtered EMG.

Then the estimate error *P*_*k*_ needs to be updated too, as follows:

(5) $$\begin{array}{r} P_{k}=\left(1-KG\right)P_{k_{p}} \end{array}$$

By changing the current state to the previous state, the algorithm is recursive to the next round:

(6) $$X_{k\;-\;1}\;=\;X_{k}$$

(7) $$P_{k\;-\;1}\;=\;P_{k}$$

The Kalman filter assumes that all errors are Gaussian-distributed. In the flowchart in Figure 2, for each measured raw EMG *Y*_*k*_, there is an output filtered EMG *X*_*k*_.

Even though Kalman filter has been applied in EMG processing (Menegaldo, 2017), the EMG model used here is different. As described above, the prediction step (Equations 1, 2) is based on our modeling on EMG. Since we aim to obtain a stable output from the noisy raw EMG, we model the real or filtered EMG signal as a constant signal. That is why the prediction of the next state *X*_*k*_*p*__ is the same as the previous state *X*_*k*−1_; this model makes the computation cost lower and the output of the filter smoother.

The *Q* and *R* values, which affect the filtering effect, were obtained by trial and error. By comparing a list of *Q* and *R* on variable raw EMG signals, we decided the *Q* and *R* with sufficient output signals, which were neither too noisy nor too delayed. In this study, the process noise variance *Q* = 0.0001, and measurement noise variance *R* = 0.59948; the same values were applied to all subjects and all channels. The control signal of the exoskeleton is obtained from the mean over eight channels of a filtered EMG signal related to the extensors of the knee (quadriceps femoris).

### 2.3. Powered Lower Limb Exoskeleton

#### 2.3.1. Actuation Design and Range of Motion

**Figure 3:**
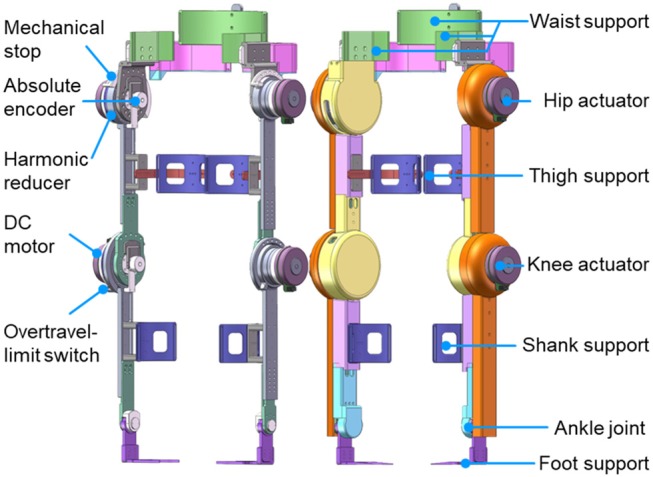
Lower limb exoskeleton without and with shell.

As shown in Figure 3, the powered lower limb exoskeleton provides active assistance at both hip and knee joints in the sagittal plane. Each active joint is driven by a brushless motor (Maxon EC 90 flat, Maxon Motor AG, Switzerland) through a harmonic reducer. The harmonic reducer (CSD-25-160-2UH, Harmonic Drive Systems, Inc., Japan) of the hip joint has a reduction ratio of 160:1 and provides a nominal joint torque of 89.6 N m, while the harmonic reducer (CSD-25-100-2UH, Harmonic Drive Systems, Inc., Japan) of the knee joint has a reduction ratio of 100:1 and provides a nominal joint torque of 56 N m. The range of motion at the hip joint is 100° in extension and 40° in flexion, while that at the knee joint is 110° in flexion and 10° in hyperextension. The ankle joint, which is in parallel with two linear springs, is a negatively adaptive joint with a range of motion from 25° in flexion to 25° in extension.

#### 2.3.2. Structure and Weight

Most supporting parts of the exoskeleton are made of aluminum, while its shell is 3D-printed. The lengths of both thigh and shank can be changed and adjusted to take account of the wearer's leg length. As shown in Figure 3, the exoskeleton is attached to the waist, thighs, shanks, and feet of the wearer. The fixation system consists of flexible bandages together with supporting connection parts on the exoskeleton, thus allowing for quick and easy fastening. The total weight of the exoskeleton is 20 kg (including the electronic components and battery), while the weight of the exoskeleton's lower leg is 0.92 kg.

#### 2.3.3. Sensing and Electronics Design

**Figure 4:**
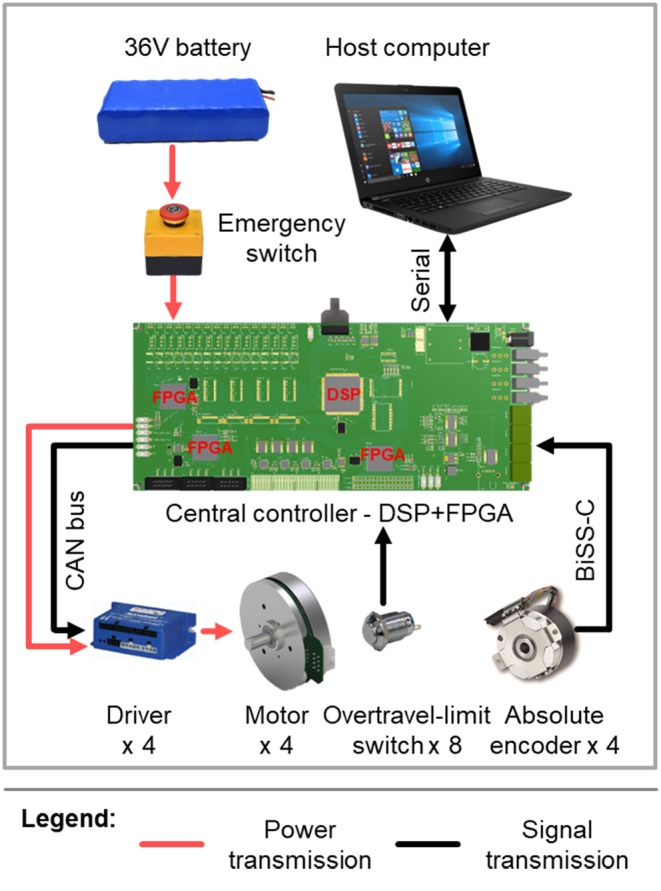
Schematic representation of the control system and the sensing and electronics design of the exoskeleton.

The joint position is measured by an absolute encoder mounted at the rotational shaft of each joint (Figure 3), whereas the joint velocity is derived from the incremental encoder that is attached to each motor. As depicted in Figure 4, real-time control is performed by a digital signal processor (DSP) and three field programmable gate arrays (FPGAs). The DSP acts as the main controller, communicating with the host computer through a serial port and sending control signals through a control area network (CAN) bus to the motor drivers. The FPGAs collect positional data from the absolute encoders through a BiSS-C interface and communicate with the DSP by parallel ports in real time.

The exoskeleton (motors and electronics) is powered by a 36 V, 6800 mA h lithium-ion polymer battery, which weighs about 0.9 kg. In this experiment, since only one knee joint was activated at one time, the exoskeleton could work for more than 3 h with this battery, which was sufficient for our experiment.

#### 2.3.4. Safety

To ensure the safety of the exoskeleton wearer, protection at three levels is implemented. The first is at the software level, limiting the moving range and moving speed of each joint in the program. The second level is electronic protection, achieved by mounting an overtravel-limit switch on each side of each active joint (Figures 3, 4). If one of these switches is pressed, i.e., if the exoskeleton has reached its limit of movement, the power is cut off. In order to prevent too much force exerted to the leg so as to hurt the user, maximum output torque of each motor is limited by the motor driver. In addition, there is an emergency switch handled by the experimenter to protect the wearer in case of emergency. The third level of protection is mechanical, preventing any joint from overtravel by means of stops (Figure 3).

#### 2.3.5. Control Algorithm

**Figure 5:**
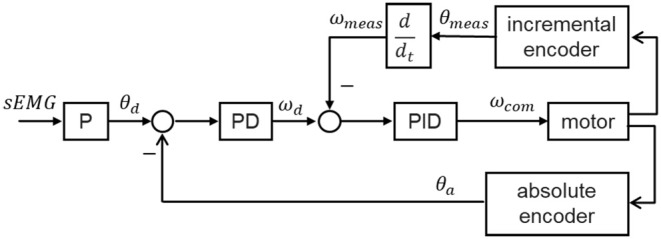
Block diagram of the control algorithm. The joint positions of the exoskeleton are proportionally controlled by the filtered sEMG signal of the subject.

As shown in Figure 5, the real-time control algorithm of the exoskeleton is based on position control with an inner velocity control loop. Proportional (P) to the filtered EMG, the desired knee joint position θ_*d*_ is sent to a proportional-derivative (PD) controller, which works as the position controller. This position controller generates the desired angular velocity ω_*d*_ according to the error between the desired joint position θ_*d*_ and the actual joint position θ_*a*_, and sends it to the velocity controller. The velocity controller using a proportional-integral-derivative (PID) control strategy is implemented in the motor driver, which acquires the motor velocity ω_meas_ from the incremental encoder and sends a control commend ω_com_ to the motor.

### 2.4. Performing the Visuomotor Training Game

**Figure 6:**
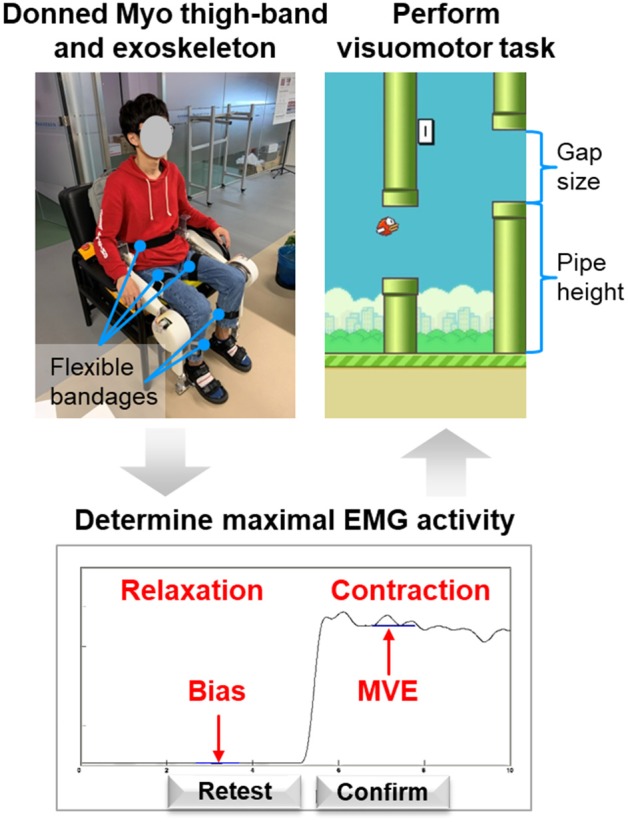
Procedure for performing the visuomotor training game.

The visuomotor training game requires the subject to sit on a chair wearing the Myo thigh-band and exoskeleton to perform knee extension movement against gravity. Before performing the training task, some preparation and calibration need to be done. The general testing procedure is depicted in Figure 6 and includes (i) donning the Myo thigh-band, (ii) donning the lower limb exoskeleton, (iii) determining the maximal-voluntary EMG range achievable by the subject, and (iv) performing the visuomotor training task. The user is provided with guidance and also visual feedback for each step. The user is allowed to repeat the procedure if necessary.

#### 2.4.1. Donning the Myo Thigh-band

**Figure 7:**
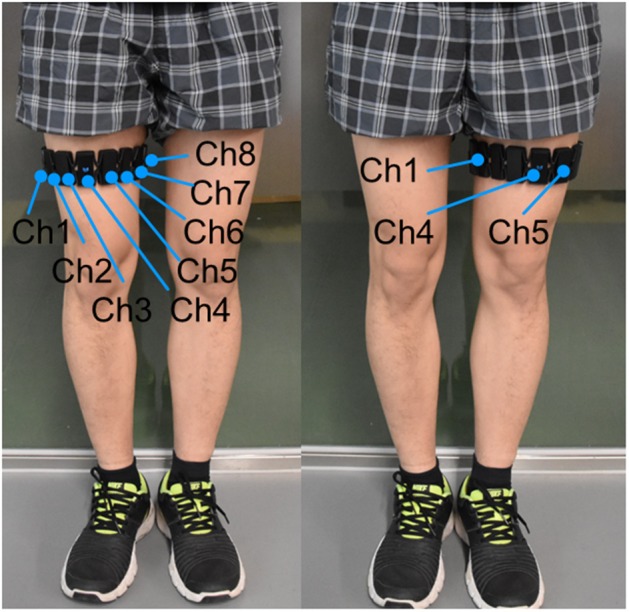
Subject with Myo thigh-band on the left or right thigh. Eight activated electrodes/channels (Ch1, …, Ch8) of the Myo thigh-band were placed on the front side of the thigh by adjusting channel 4 (Ch 4) with a blue flashing light in the middle of the front thigh.

The extensors of the knee (quadriceps femoris muscle, etc.) are the muscles most significantly involved in knee extension against gravity, whereas the activation level of the flexors (biceps femoris muscle, etc.) is very low (Chen X. et al., 2017). This is because in such voluntary movements, the subject can flex his or her knee joint under the force of gravity without activating the flexor muscles (Chen X. et al., 2017). Therefore, we collected EMG data only for the knee extensors. The subject was instructed to put the Myo thigh-band on the middle of right or left thigh (around 200 mm relative to the knee joint, where the rectus femoris located; Figure 7). During the test, only half of the electrodes of the thigh-band were activated, namely, eight channels on the quadriceps femoris side of the thigh. Therefore, when donning the Myo thigh-band, the activated part (with a blue flashing light) was placed on the quadriceps femoris side (front side) of the thigh.

#### 2.4.2. Donning the Knee Exoskeleton

The exoskeleton was placed on a comfortable chair with the hip joints fixed at 90° in extension and the knee joints initialized at 90° in flexion (Figure 6). The subject just needed to sit on the chair and fix their shanks, thighs, and waist to the supporting parts of the exoskeleton. The experimenter provided any necessary assistance to the subject. In this setup, only one knee joint of the exoskeleton was activated and could be controlled by the sEMG of the subject (Figure 5), whereas the other three active joints were fixed at the initial joint angles.

In the following description, a knee joint angle of 0° represents the sitting posture, i.e., with the thigh perpendicular to the shank, while a knee joint angle of 90° means that the thigh and shank are in the same line.

#### 2.4.3. Determining Maximal EMG Activity

After the subject had donned the Myo thigh-band and the exoskeleton, their maximal EMG activity was determined (Figure 6). The signal using here was the mean over eight channels of the filtered EMG related to knee extensors. During this process, the subject first relaxed for 5 s and then performed knee isometric contraction for 5 s with the exoskeleton on. We determined the maximal-voluntary EMG (MVE) that could be maintained for at least 1 s with maximal voluntary contraction of knee extensors. Similarly, the bias (Bias) was obtained from the measured EMG signal when the muscles were relaxed. Since patients in the post-stroke sensitive period are also unable to produce overt movement, testing MVE with isometric contraction is meaningful for both stroke patients and healthy subjects. The value obtained was used to adapt the knee exoskeleton movement individually to the EMG range of the subject. In other words, the knee joint angle was proportional to the filtered EMG, with Bias and MVE being related to the minimum (0°) and maximum (90°) knee joint angles respectively. To avoid fatigue, only 60% of the maximal EMG activity was applied for the below training task, which means that the knee joint movement range was from 0° to 54°.

#### 2.4.4. Flappy Bird Visuomotor Training Task

Previous studies shows that task-oriented intense training in an environment that provides timely feedback, motivation, stimulation and confidence significantly improves rehabilitation outcome (Johansson, 2011). Videogame-based intervention with these features has attracted attention. Figure 6 shows a screenshot of the Flappy Bird visuomotor training task, which depicts a bird flying in the sky with some pipes as obstacles. The task aims at improving knee joint movement stability and accuracy, as well as thigh extensor muscle strength against gravity. It also has potential to facilitate motor recovery and provide new possibilities for cortical reorganization and enhancement of functional mobility (Santos et al., 2016). The goal of this task is to control the bird's flight across the pipes. The position of the flappy bird in the vertical direction on the screen is proportional to the knee joint angle of the exoskeleton, which means that the lowest and highest positions of the bird in the sky correspond to knee joint angles of 0° and 54°, respectively. All subjects were explained and guided how to use their muscles (or EMG) to control the exoskeleton or the bird. Extension movement or bird flying up can be achieved by activating their thigh extensors; flexion movement or bird flying down can be achieved by relaxing their thigh extensors.

In each block, the subject had four bird lives (or trials). If the bird flew across a pair of pipes (upper and lower pipes), the subject gained one point as a reward. However, if the bird hit the pipes, the subject would lose one bird life and the bird would hover on the sky and stop moving forward. When the subject was ready for the next flight, he or she could press the space key to start the next bird life and move forward again. The subject's aim was to obtain as many points as possible with four bird lives. Once all four lives had finished, the game stopped and the subject could choose either to exit or to replay the game. To prevent fatigue, subjects were provided with rest intervals throughout the experiment (Video S1).

Multisensory (vision, audio, touch) feedback to the subject about the movement performance was achieved (Figure 1). Different sounds indicating gaining one point or losing one bird life were played along the game. Haptic feedback generated from the vibrating motor of the Myo thigh-band worked as a punishment when the bird hit the pipes. Such enriched game experiences have been demonstrated to increase patients' motivation and facilitate functional recovery by engaging appropriate neural circuits in the motor system (Perez-Marcos et al., 2017).

To make the game more interesting and challenging, some of its parameters were adjusted during the process. The pairs of pipes appeared randomly at different heights, and the gap between the pipes in each pair became narrower as the score increased. At the same time, the bird's flying speed in the horizontal direction also increased with the score. Both the narrowing gap and increasing flying speed made the game become more and more difficult, which meant that the subject needed not only to move the knee joint in a more stable manner, but also to respond to the changes in the pipes more quickly. Neurorehabilitation programs should include activities or tasks that enable patients to float in their Flow Zone, defined as where the person is at a high level of enjoyment with a balance between the difficulty of the task and the abilities of the person (Perez-Marcos et al., 2018). Following this principle, the Flappy Bird game tried to make the subject feel comfortably challenged and highly engaged by the task. Maintaining a state of flow is important for promoting patients adherence to treatment, especially for home-based rehabilitation (Perez-Marcos et al., 2018).

**Figure 8:**
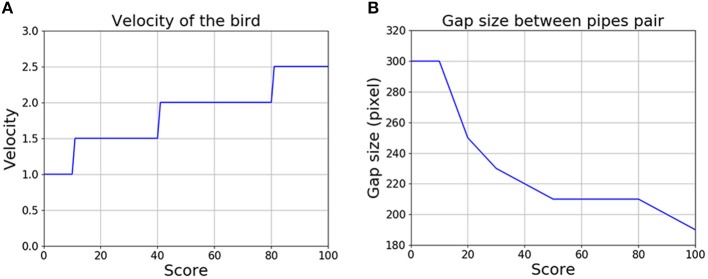
The game becomes more challenging as the score increases. **(A)** Change in the velocity of the bird. **(B)** Change in the gap between the upper and lower pipes in each pair.

Figure 8 shows how the difficulty of the game changed, as represented by the increasing bird velocity and decreasing gap size between pipes, as the score increased. The velocity of the bird in the horizontal direction was low at first and increased gradually to 2.5 times its initial value. At the same time, the gap size decreased from 300 pixels to 190 pixels (the size of the bird remained at 48 pixels throughout). Once the score exceeded 100, the level of difficulty ceased to change. The low initial speed and relatively wide gap between pipes allowed the subject to practice and learn the game at the beginning. Once the subject had become familiar with the control, the game became more and more challenging, which also provided some motivation for the subject to continue to play.

### 2.5. Experimental Design

Six healthy subjects were recruited to take part in the experiment to investigate whether they could use the EMG-controlled knee exoskeleton to assist them in home rehabilitation and to further improve the EMG control strategy with repetitive task training. Each subject performed a 10-block visuomotor training game with each leg, with interblock rest intervals of 30 s. In order to control for leg dominance, subjects were randomly assigned to two groups. Half of the subjects started with the left leg and the other half with the right leg. After finishing the game with one leg, they transferred the Myo thigh-band to the other leg and continued.

**Figure 9:**
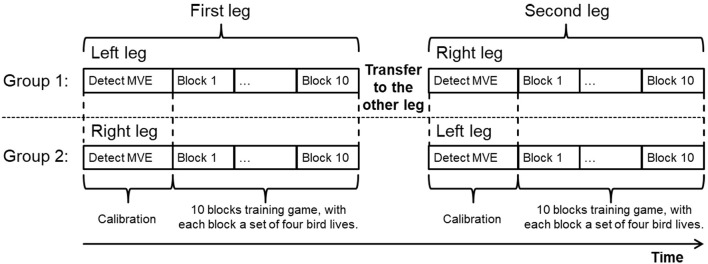
Experimental protocol. Subjects were assigned to two groups, with one group (three subjects) starting with the left leg and the other group (three subjects) with the right leg. All subjects performed the training task with both legs. For each leg test, after determining MVE, 10 blocks training game was performed, with each block consisted of four bird lives.

The experimental protocol is shown in Figure 9. Since all the subjects were naive to the game, the experimenter first explained the game to them and then guided them in donning the Myo thigh-band and exoskeleton. After the MVE had been detected, the subjects played the game themselves. The experimenter sat beside the subject during the whole testing procedure with the emergency switch in hand in case of emergency and also to provide any guidance needed. One session test, which included 10 blocks training on the left leg and 10 blocks training on the right leg, lasted around 75 min.

### 2.6. Evaluation Metrics

#### 2.6.1. Score

The score is the points obtained by the subject within one block. As the main evaluation variable of this visuomotor game, we further calculated the mean and standard deviation (SD) of the score across six subjects for both the first and the second legs.

#### 2.6.2. Muscle Activation Level

In order to quantify how much the subjects actually activated their muscles, we defined the muscle activation level (*MAL*) at each time step as

(8) $$0\leq MAL(t)=\;\frac{EMG(t)\;-\;Bias}{MVE\;-\;Bias}\leq 1,$$

where *EMG*(*t*) was the processed EMG signal extracted from the thigh extensors. The parameters *MVE* and *Bias* were measured during the calibration process. Then, we can obtain mean muscle activation level (*mMAL*) in each block via

(9) $$mMAL\;=\frac{\sum_{t\;=\;0}^{T}MAL(t)}{T},$$

where *T* was the total time steps in one block. Similarly, we also calculated the mean and SD of *mMAL* across six subjects for both the first and the second legs.

#### 2.6.3. Block Activation Time

The block activation time (BAT) is the time taken for the subject to actively play one block of the Flappy Bird game. Block activation time represents active therapy duration provided to the subject. As an important metric reflecting the therapy dose or intensity, it is a critical factor to achieve a positive outcome. The mean and SD of block activation time across six subjects for the each leg were calculated.

#### 2.6.4. Statistical Analysis

A one-way analysis of variance (one-way ANOVA) was performed when appropriate for the above metrics.

## 3. Results

To evaluate the performance of the training system as well as the subjects, we analyzed the data from the experiment, with the following results.

### 3.1. Time to Set Up the Training

For home rehabilitation, it is important to simplify the setup process, since the therapist cannot be present at each session. With the Myo thigh-band, the electrodes do not need to be placed precisely over specific muscles. The subject just needs to don the thigh-band with the activated part on the front of the thigh by themselves or with the assistance of anyone around. The only calibration procedure required is to determine the MVE automatically with the participant performing according to onscreen guidance. One participant was asked to set up the training independently 10 times, and the time spent was measured. According to this test, the entire setup process, including donning the Myo thigh-band and knee exoskeleton and determining MVE, took 73.2 ± 10.7 s.

### 3.2. Performance of the Kalman Filter

**Figure 10:**
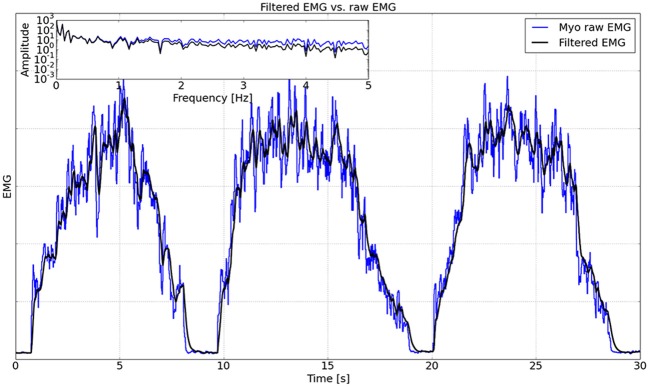
Comparison between raw EMG and filtered EMG. The inset shows the FFT analysis on a logarithmic scale up to 5 Hz.

To quantify the performance of the Kalman filter, one subject was asked to activate the muscle extensors three times in 30 s. The raw Myo EMG data from one channel measuring the extensors and the corresponding EMG data filtered using the Kalman filter are shown in Figure 10, from which it can be seen that the filtered EMG is much smoother than the raw EMG. As shown in the inset of Figure 10, a fast Fourier transform (FFT) was performed to analyze the frequencies of the raw and filtered EMG data. The FFT analysis demonstrated that the Kalman filter attenuated signal noise at frequencies above 1 Hz in the raw EMG. We implemented a cross-correlation analysis (Cohen, 2014) on the raw and filtered EMG data, and the result showed that the delay caused by the Kalman filter was 64 ms.

### 3.3. Performance of the Knee Exoskeleton

Since the control strategy of the knee exoskeleton is based on position control, the most important evaluation criterion is its tracking ability (Jia, 2000), which can be quantified by the root mean square error (RMSE) between the desired joint angle and the actual joint angle.

**Figure 11:**
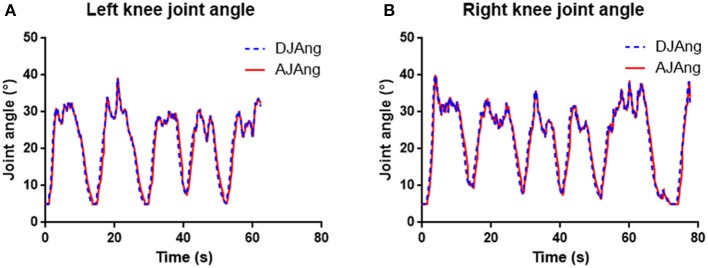
**(A,B)** Left and right knee joint angle tracking performance of the exoskeleton from one subject in one typical trial of the experiment. Blue dash lines show the desired joint angles (DJAng), and red solid lines represent the actual joint angles (AJAng).

Figure 11 shows the typical knee joint angle tracking performance of the exoskeleton, based on data obtained from one subject in this experiment. The desired joint angle is proportional to the filtered EMG signal (the mean over the eight channels of the filtered EMG) and the actual joint angle is measured by the absolute encoder at the knee joint. In this figure, the lower values (<10 °) are the relaxed phase, while the higher values (>20 °) indicate how the participant controlled the bird to cross the pipes in the game. We can see that the actual joint angle of the exoskeleton generally moved along the desired joint angle.

We analyzed the data (10 blocks for left legs and 10 blocks for right legs) from all six subjects performing the Flappy Bird game, considering two legs separately or both together, and the results are shown in Table 1. As can be seen, the RMSE between the desired and actual joint angles was 1.56° ± 0.21° when we considered both legs together. A one-way analysis of variance (one-way ANOVA) was performed in Python and further indicated that there was no significant difference between left (1.54° ± 0.22°) and right (1.59° ± 0.19°) legs on the RMSE metrics (*p* = 0.706 > 0.05).

**Table 1:** RMSE and time lag of the exoskeleton across subjects.

**Leg**	**RMSE (deg)**	**Time lag (ms)**
Both legs	1.56 ± 0.21	110 ± 32
Left legs	1.54 ± 0.22	108 ± 28
Right legs	1.59 ± 0.19	122 ± 36

*This table displays the mean and standard deviation of RMSE and time lag of the exoskeleton across subjects. All six subjects, using either left legs or right legs, were analyzed*.

The time lag between the desired and actual joint angles was calculated using a cross-correlation analysis (Cohen, 2014), and the results are also presented in Table 1. The time lag caused by the exoskeleton was around 110 ms and there was no significant difference between left and right legs in terms of time lag (*p* = 0.864 > 0.05) according to one-way ANOVA.

### 3.4. Performance of the Subjects

#### 3.4.1. Score

**Figure 12:**
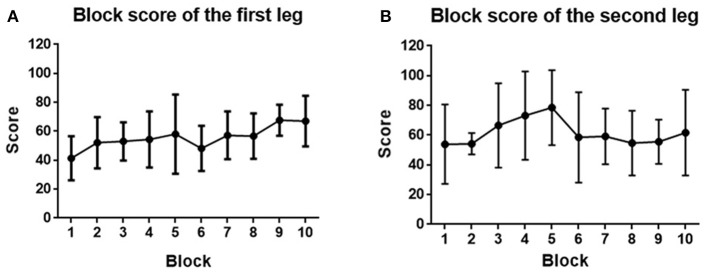
**(A)** Block score averaged across the first leg of subjects. **(B)** Block score averaged across the second leg of subjects. Filled points show the mean scores, and error bars represent the standard deviation.

We quantified the performance of the subjects by the scores they obtained in each block. Since each subject took part in the game using both legs in turn (either left and then right or vice versa), we analyzed the performance of the first and second legs to be tested. As can be seen in Figures 12A,B, for the first leg, the performance generally became better and better (score from 41.3 ± 15.2 to 67.0 ± 17.4) and reached its highest level at the end of the game. However, for the second leg, the performance was initially better (a starting score of 53.8 ± 26.7), and rose quickly to give the highest score (78.5 ± 25.2) at block 5, after which it deteriorated and then generally kept stable until the end of the game (61.7 ± 28.7). It can be seen that for both the first and second legs, the score improved after 10 blocks training, even though no significant difference was found (*p* > 0.05). This may be because the game became more and more challenging with the score increasing, which means gaining one more point at the end is much harder than at the beginning.

#### 3.4.2. Muscle Activation Level

**Figure 13:**
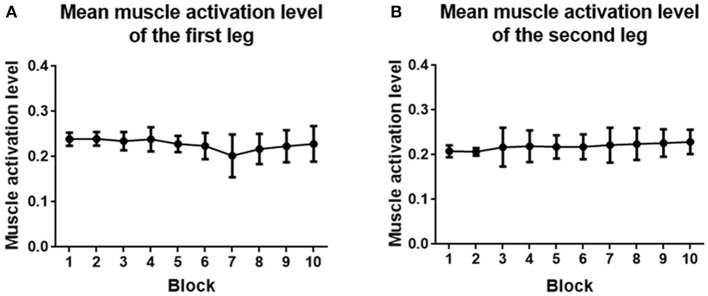
**(A)** Mean muscle activation level averaged across the first leg of subjects. **(B)** Mean muscle activation level averaged across the second leg of subjects. Filled points show the mean *mMAL*, and error bars represent the standard deviation.

Muscle activation level analysis showed that *mMAL* generally kept stable between 20% and 30% during the whole training process (Figure 13). One-way ANOVA indicated that there was no significant difference between each block (*p* > 0.05) for both legs.

#### 3.4.3. Block Activation Time

**Figure 14:**
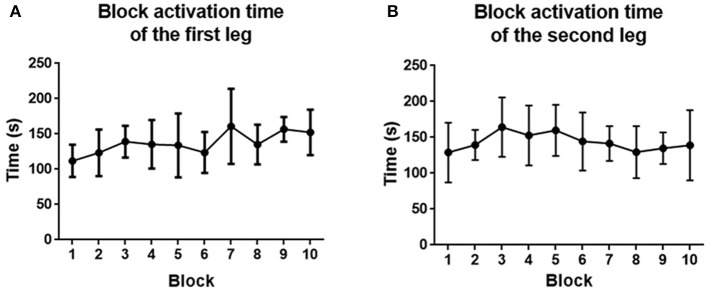
**(A)** Block activation time averaged across the first leg of subjects. **(B)** Block activation time averaged across the second leg of subjects. Filled points show the mean block activation time, and error bars represent the standard deviation.

The block activation time of the first and second legs is depicted in Figure 14. For the first leg, the block activation time increased from 111.8 ± 22.8 to 152.4 ± 32.2 s. For the second leg, the block activation time increased from 129.0 ± 41.5 to 139.1 ± 48.89 s. No significant improvement was found at the end of the training for both legs (*p* > 0.05).

## 4. Discussion

The observation or imagination of body movements facilitates motor recovery and provides new possibilities for cortical reorganization and enhancement of functional mobility (Santos et al., 2016). Thus, it appears that movement visualization may play an important role in motor rehabilitation (Santos et al., 2016). Motor recovery of stroke patients who are too weak to make overt movements is a big challenge, since voluntary muscular contractions do not lead to significant sensory feedback, which makes rehabilitation training less effective in motivating and enhancing motor skill learning gains (Pereira et al., 2015). The use of EMG-controlled exoskeletons together with visuomotor training tasks might provide a new opportunity for this group of patients. Nevertheless, previous studies of EMG control found low predictability and high variability, which may impair motor learning.

In the present paper, a preliminary study was conducted and we found that healthy subjects could learn to control a user-friendly knee exoskeleton to perform an interesting visually guided game using EMG signals in a simulated home setting. The setup was significantly simplified by improving the system in a number of ways, such as reassembling the EMG electrodes and introducing a new signal processing method, thereby making it possible to assist patients undergoing home rehabilitation. The proposed home-based rehabilitation system should allow improvements in the intensity of training and make rehabilitation more convenient for the patient. The results further indicated that all subjects had better task performance through training. Initial feedback from voluntary subjects confirmed that this interesting and challenging training system is not only easy to use but also provides motivation for the patient, making it a promising strategy for active training of patients in the early rehabilitation phase.

### 4.1. EMG Controller and Knee Exoskeleton: System Characteristics

By combining two Myo armbands, the Myo thigh-band used here provides a more convenient instrument to acquire EMG data online compared with traditional EMG systems (see, e.g., Wolf and Binder-Macleod, 1983; Armagan and Oner, 2003; Song and Tong, 2005; Crow et al., 2009; Buongiorno et al., 2016; Peternel et al., 2016; Ao et al., 2017; Chen X. et al., 2017; Irastorza-Landa et al., 2017; Yun et al., 2017). In particular, both patients and healthy subjects could don and remove the device easily without the therapist being present because of its use of dry electrodes and expandable flex. By guiding the subject to perform one-knee isometric extending contraction at a maximum level through a calibration routine, the training system is individualized, which provides an intrinsically adaptive aspect when the training lasts several days or even weeks. The short setup time, with a calibration process taking only about 73.2 s, makes this system greatly appreciated by users.

Another challenge facing EMG-based control systems is the need to transform highly variable raw EMG into a smooth, rapid response control signal. Since delays can impair visuomotor control and learning (Honda et al., 2012), here we used a Kalman filter to remove signal noise above 1.2 Hz with a time lag of 64 ms. Because the knee extension involved almost all the muscles on the quadriceps femoris side, our control signal used the mean over eight channels of the filtered EMG on the extensor side of the thigh.

The knee exoskeleton here acts like a therapist, providing assistance to patients in their rehabilitation by performing a specific task, but with control and intention provided by the patients themselves. The exoskeleton has the potential to assist stroke patients to move their lower legs as they wish so that movement visualization can be achieved. The time lag caused by the exoskeleton was about 110 ms.

Previous studies have shown that electromechanical delay (EMD), which is typically defined as the time lag between electrical activation of a muscle and the onset of the exerted force (Cavanagh and Komi, 1979), is between 30 and 150 ms (Zhou et al., 1995; Blackburn et al., 2009; Nordez et al., 2009; Yavuz et al., 2010). Úbeda et al. (2017) even found EMDs ranging from 112 to 361 ms. Considering that EMG appeared about 125 ms before force generation (Blackburn et al., 2009), the 170 ms time lag in our training system (which was caused by both the filter and the exoskeleton) is very short and acceptable. Most subjects in our experiment said that they did not feel any time lag in the system.

### 4.2. Flappy Bird Game

For stroke rehabilitation, motivation is especially important. Studies shows that motivation influence on the effectiveness on rehabilitation (Rapoliene, 2018). Activating patient participation in the therapy is a guiding principle of rehabilitation (Sitaram et al., 2016). The Flappy Bird game not only makes the rehabilitation process more interesting, but also motivates the patient to take an active part in the training. Making the game neither too easy nor too difficult for patients is key: that is to say, the game should be fitted to the patients, rather than the other way round. By adjusting the difficulty to the patient's pace of recovery not only maximizes training potential, but also prevents habituation and frustration (Perez-Marcos et al., 2018). Enable patients to float in their Flow Zone facilitates keeping patient motivation at an optimal level during the long rehabilitation process (Perez-Marcos et al., 2018). In our experiment, we started the task with a low level of difficulty and gradually made the task become ever more challenging. This allowed the subjects to learn and adjust to the task at the beginning and then improve their skill gradually as the difficulty increased.

Rehabilitation dose, which might be a critical factor to achieve a positive outcome, can be also increased since this challenging game can motivate subjects to continue with rehabilitation with the aim of improving their scores in the game. Appropriate and timely feedback (e.g., reward and punishment) together with adaptation of difficulty levels can boost and maintain patients' motivation as long as possible (Perez-Marcos et al., 2018). Besides positively affecting motivation and enjoyment of training, videogames also impact cognition. In particular, playing action videogames (i.e., games that emphasize physical challenges) has been shown to robustly enhance attention and spatial cognition (Perez-Marcos et al., 2018).

Brain plasticity is the base of rehabilitation (Johansson, 2000). Closed-loop neurofeedback and real-time training is good for brain plasticity (Sitaram et al., 2016). As shown in Figure 1, the flappy bird in the game, the EMG controller, and the knee exoskeleton together with the subject form a closed control loop. In this loop, playing the game to obtain as many points as possible becomes the objective of the patient. This makes the patient generate intentional movement. The Myo thigh-band records the EMG signals, and the EMG controller decodes these to produce the intended knee movement. By actuating the motor, the patient can perform the desired movement with the assistance of the knee exoskeleton. With this complete loop, we actually change the objective of stroke patients from doing rehabilitation exercises to playing an interesting game. All the movements in the loop are actively performed by the patients themselves. By involving the corticomotor system in the training process, we may make rehabilitation more effective.

There is substantial evidence that the post-operative environment can influence the outcome after stroke (Jess and Hannan, 2006). Enriched environment and rehabilitation augments neuroplastic processes and compensates neuronal growth, which ultimately contributes to improved motor function and cognitive skills (Biernaskie and Corbett, 2001). Multisensory simulation from the videogame provides patients with enriched rehabilitation, which is able to evoke the mirror neuron system and mechanisms of action observation (Perez-Marcos et al., 2018). In the Flappy Bird game, not only visual, but also auditory and haptic feedbacks were implemented. It involves subject's auditory nervous system and haptic perceptual system into the training process. Multisensory stimulation, challenging gaming environment, incorporating closed-loop mechanics can boost the rehabilitation effect (Perez-Marcos et al., 2018).

### 4.3. Skill Acquisition by the Subjects

In the visuomotor training task, the randomized pipe height requires the subjects to voluntarily activate and maintain their levels of muscle excitement, while the variation in the gap between pipes demands that the subjects actively control their level of muscular accuracy. An improved final score in each block indicated that subjects improved their control skill during one training session. The performance with the second leg, which exhibited not only a higher starting score and but also the highest overall score, was generally better than the performance with the first leg. This indicated that healthy subjects could shift their learned skill from one leg to another. However, it remains to be seen if the same would be true in stroke.

The activation level of the muscles did not change too much during training. This is because the game setting is similar for each block. By changing the knee joint movement range, the muscle activity can be affected. However, in order to quantify the score in the same difficulty level, we did not change it in this experiment. The block activation time relating to the rehabilitation dose, was improved. However, no significant effect was found, which might be caused by the short training period. By increasing the training sessions, the block activation time has potential to improve more.

In this study, we quantified how much the participants actually activated their muscles, and the timing of that activation. Task performance and training dose were improved even though the training period was quite short. By increasing the training period, significant improvement is possible.

### 4.4. Limitations and Future Directions

Even though we believe that the proposed EMG-controlled exoskeleton training system has the potential to enhance stroke rehabilitation outcomes, several potential problems still need to be considered before it can be adopted for use with patients. First, although the sample rate of the Myo thigh-band and the filtered EMG signal quality were adequate for healthy subjects, more test needs to be done to decide whether this device and the filter method are appropriate for stroke patients. By applying this device and filter method to visual feedback tasks like EMG based target tracking, we may be able to collect EMG data from stroke patients. We can further prove whether the filtering method working on them. Second, adapting the difficulty of the game individually to keep patients motivated may increase the acceptability of the system by patients. Since gamified tasks can also help strengthen brain modulation, adapting the training based on the patient's needs and performance can make the rehabilitation program more effective (Perez-Marcos et al., 2018). Third, the range of motion, which also influences the muscle activation level during training, will also be individualized for stroke patients. In addition, although the knee exoskeleton could be proportionally controlled by healthy subjects, there is still concern regarding whether this will be the case for stroke patients, especially given the possible risk of additional injury during training. For example, patients' unwanted muscle activity like spasticity may also cause the exoskeleton to move proportionally, which may hurt the patients. More safety measures should be taken and tested. Finally, given the knee flexion movement also involved knee flexors, it is worth to test whether collecting knee flexors EMG and introducing it to the control could lead to a better performance. Whether the proposed training system can be realized in stroke patients with very weak muscle activity is something that will be tested in the future.

## 5. Conclusion

This paper has described the development and evaluation of a rehabilitation system for home use that combines an EMG-controlled exoskeleton driven by knee extensors and an interesting visuomotor game that provides motivation for patients. By overcoming a number of difficulties, we have made it possible for the system to be used without the need for a therapist to be present at each session, thus significantly decreasing the cost of training and increasing the intensity and outcome of the rehabilitation process. Initial testing in healthy subjects suggests that using the EMG-controlled exoskeleton in a game context to carry out rehabilitation is possible and that the training system facilitates learning of motor skills. Further tests need to be done on stroke patients with low muscle activity to determine whether the EMG-controlled exoskeleton and visuomotor training task implemented here are suitable for them. A user-friendly home rehabilitation tool like this may improve the outcomes of rehabilitation for patients in the initial rehabilitation phase.

## Data Availability

The raw data supporting the conclusions of this manuscript will be made available by the authors, without undue reservation, to any qualified researcher.

## Ethics Statement

The study was approved by the Biological and Medical Ethics Committee of the Beijing University of Aeronautics and Astronautics in accordance with the Declaration of Helsinki, and all subjects gave written informed consent before participation.

## Author Contributions

ML, W-HC, and XD were responsible for the study conception and designed the experiment. ML and W-HC developed the exoskeleton and the EMG-based training system. ML conducted the experiment and collected the data. ML, W-HC, XD, JW, and ZP analyzed the data. ML, W-HC, and XD wrote the paper. BZ contributed to manuscript preparation. All authors corrected several versions of the paper and approved the final manuscript.

### Conflict of Interest Statement

The authors declare that the research was conducted in the absence of any commercial or financial relationships that could be construed as a potential conflict of interest.

## Supplementary Material

Video S1 An experimental subject wearing the EMG-controlled exoskeleton to play the Flappy Bird game.
